# The Crosstalk between Microbiome and Immunotherapeutics: Myth or Reality

**DOI:** 10.3390/cancers14194641

**Published:** 2022-09-24

**Authors:** Alireza Tojjari, Hassan Abushukair, Anwaar Saeed

**Affiliations:** 1 Department of Medicine, Division of Medical Oncology, Kansas University Cancer Center, Kansas City, KS 66205, USA; 2 Faculty of Medicine, Jordan University of Science and Technology, Irbid 3030, Jordan

**Keywords:** cancer, immunotherapy, microbiome, immune checkpoint inhibitor, immunology, therapy

## Abstract

**Simple Summary:**

The gut microbiota can mediate the balance between human health and disease, making the microbiome a critical organ. The gut microbiota can locally and systemically regulate the host’s immune system. Cancer immunotherapy has evolved as an essential method for treating cancer patients. Rapidly evolving data suggest that the microbiota influences the therapeutic efficacy of immunotherapy, such as immune checkpoint inhibitors. However, the specific effect of the gut microbiota on immunotherapy-treated malignancies remains unclear, and multiple reports have been released with conflicting results. The association between the gut microbiota with cancer immunology and immunotherapy is discussed here, with an emphasis on the relationship with immunotherapy outcomes.

**Abstract:**

The gut microbiome refers to microorganisms and their genetic material influencing local and systemic inflammation. Inflammation is known to contribute to cancer development, progression, and treatment. Evidence suggests that modulating the gut microbiome may affect responses to various cancer therapies. The gut microbiota has been suggested to have an impact on immunotherapy efficacy, especially the currently widely used immune checkpoint inhibitors in various malignancies. Microbial interventions like fecal microbiota transplantation, various probiotics, or even antibiotics can increase or decrease the tumor’s sensitivity to immunotherapy. However, not all tumors react in the same manner, highlighting the tumor microenvironment heterogeneity across tumor types and the influence this has on the crosstalk between the microbiome and therapy outcomes. In this study, we intend to review the association between the gut microbiota and immunotherapy response in cancer patients and the factors regulating this interaction.

## 1. Background

The highly regulated microbiota within the gastrointestinal system is one of its most critical functioning components [1,2]. These microbes have coevolved with humans to perform several roles that are beneficial to human health, including extracting unavailable nutrients from particular foods and contributing to the growth and stability of the immune system by maintaining the integrity of mucosal barriers. Our understanding of the microbiome has grown with the development of high-throughput sequencing methods over the last decade [3,4]. The gut microbiota regulates the balancing act between inflammation, infection, and tolerance to food and food antigens, and plays a crucial role in innate and acquired immune responses. The gut microbiome has a systemic function in the body and impacts the intestine and local immune physiology [5,6]. The human microbiome comprises ~3 × 10^13^ bacteria; most of them are commensals [7]. Human diseases are intricately bound to the microbiome. The negative modulation of the gut microbiome (dysbacteriosis) has been linked to various digestive, neurological, and endocrine disorders. In addition, bacterial and viral infections have been linked to carcinogenesis and the efficacy and toxicity of cancer therapy [8,9,10,11,12,13,14]. The 2013 Breakthrough of the Year was given to cancer immunotherapy based on therapeutic developments in two categories: chimeric antigen receptor (CAR)–modified T cells, and immunological modification using antibodies to suppress immunological regulatory checkpoints. In the past few years, immunotherapy targeting immunological checkpoints has led to considerable improvements in patient prognosis in a variety of malignancies, with agents such as Programmed Cell Death 1 (PD-1)/Programmed Cell Death-Ligand 1 (PDL-1) inhibitors and Cytotoxic T Lymphocyte-associated Antigen-4 (CTLA-4) inhibitors [15]. According to emerging evidence, the gut microbiome plays a vital role in influencing the effectiveness and toxicity of cancer immunotherapy. For instance, it has been found that Ruminococcaceae correlate with the therapeutic advantages of anti-PD-1/PD-L1 treatment, and B. fragilis, B. thetaiotaomicron, and Burkholderiales are associated with anti-CTLA-4 efficacy [16,17,18].

Additionally, fecal microbiota transplantation (FMT) from responsive patients to germ-free animals boosted the anticancer efficacy of anti-PD-1 therapy [19]. Due to its close interaction with the immune system, the gut microbiome has received growing interest for its potential role in modulating cancer immunotherapy effects [20,21]. Recent findings from various research papers have attempted to address this topic, yet controversial outcomes made it challenging to draw a conclusive relationship. While the use of antibiotics preceding or concurrently with immune checkpoint inhibitors (ICIs) may adversely affect anti-tumor responses and survival in certain types of malignancies, a favorable impact is seen in other malignancies [22]. Some phylum has a significant correlation with positive results in ICIs treated tumors, such as Firmicutes and Verrucomicrobia, while others, such as Proteobacteria, have negative impacts, and still others, such as Bacteroidetes, have mixed impacts [23]. A recent review highlighted the potential role of the gut microbiome as a predictive biomarker for clinical response and adverse events in cancer patients on ICIs. By including only clinical studies, they reported a substantial difference in the gut microbiome composition between ICI responders and non-responders, specifically three out of the nine included studies showed that the abundance of Firmicutes and Actinobacteri bacteria was associated with ICI response [24]. In this review, we provide a comprehensive look into the landscape of the gut microbiome and immunotherapeutics. We shed light on the supporting data for the microbiome’s importance in regulating tumor immune mechanics and how it contributes as one of the dictators of ICIs efficacy. In addition, we discuss the potential role of antibiotics in modulating immune responses to ICI.

## 2. The Microbiome and the Immune System

Gut microbial composition is associated with several factors, including the method of a child’s birth, the composition of maternal microbiota, genetics, lifestyle, drugs, supplementarity, and environmental factors [25,26]. The microbiome and the immune system interact continuously at various sites throughout the body. The gut microbiota plays several critical roles in host defense [7,27]. Additionally, commensal bacteria and gut-associated lymphoid tissue interact closely and stimulate B and T cell differentiation, maturation, and activation [28,29,30,31,32]. By controlling the growth of Tregs and Th17 cells, the gut microbiota could keep immunological tolerance and inflammatory response in a homeostatic balance.

On the other hand, dysbiosis, an imbalance or disturbance in the environment, destroys the balance of microorganisms in the gut. The development of opportunistic pathogens is indicative of dysbiosis, characterized by an imbalance or reduction in the quantity, diversity, and stability of microorganisms [33]. Various bacteria assist in the battle against cancers by stimulating immunity, whereas others mediate immunosuppression, allowing cancer cells to evade the immune system [34].

## 3. The Impact of Microbiome on Cancer and Cancer Therapeutics

There is growing evidence that the variety and composition of gut bacteria influence the therapeutic efficacy of various cancer treatments [19,35,36,37,38,39,40,41,42]. In 1910, Coley WB, for the first time, injected streptococcal organisms into a patient suffering from unresectable sarcoma, resulting in excellent antitumor responses [43]. In addition, urinary bladder cancer is the only malignancy treated with a living microorganism, Mycobacterium Bovis bacillus Calmette-Guérin (BCG). Therefore, the idea that certain bacteria may protect against the development of malignant disease is evident at this point. Although it has been used for over four decades, the molecular complexities of its therapeutic effects are not fully known. One theory proposed that BCG binds to urothelial cells, which was subsequently followed by the internalization of bladder cancer cells and the development of cytotoxic immune responses that destroyed malignant tissue [44]. Another study revealed that Enterococcus hirae could be used to compensate for tumor dysbiosis [45]. Other therapeutic methods, like chemotherapy, also closely interact with the microbiome. For instance, they can promote the growth of species like Bacteroides, Escherichia, and Enterococcus faecium, while preventing the growth of Clostridium IV and Clostridium XIVa, Firmicutes, Veillonella, Faecalibacterium prausnitzii, Bifidobacterium and Lactobacillus species [46,47]. The 5-FU regimens enhanced the proliferation of facultative gram-negative and anaerobic bacteria in the oral cavity and the gastrointestinal system, respectively [48]. The activation of signaling pathways and innate immune components in the gut is important for the maintenance of the barrier function by protecting the gut from damage, and stimulating mucosal restoration. Chemotherapy changes microbial homeostasis by inhibiting commensal bacteria proliferation, which results in detrimental effects on barrier functionality, intestinal integrity, and repair pathways activation [49]. Other anti-cancer modalities like radiation can also modulate or interact with the microbiome; for example, there is a known correlation between the changed oral microbiota of nasopharyngeal cancer patients and more severe radiation-induced mucositis. After irradiation, the prevalence of Streptococcus mitis in patients with nasopharyngeal cancer rose considerably [50]. Patients with radiation-induced diarrhea demonstrated a higher alteration in the gut flora than their counterparts. Bacteroides, Dialister, Veillonella, and unclassified bacterial species rose, whereas Clostridium XI and XII, Faecalibacterium, and Oscillibacter, Parabacteroides, and Prevotella decreased [47,51]. Additionally, a high prevalence of Clostridium difficile infection and a high mortality rate were seen in patients who underwent radiotherapy [52].

## 4. The Microbiome’s Impact on Immune Modulators Efficacy

Immunotherapy is a comparatively modern treatment that has become a very effective treatment method for solid tumors. The two established ICI targets are cytotoxic T-lymphocyte associated protein 4 (CTLA-4) and programmed death receptor and its ligand 1 (PD-1/PD-L1). The connection between microbiome composition and ICI efficacy has been highlighted in several previous clinical and preclinical studies. For the first time in 2015, Sivan et al. found an interaction between gut microbiota and immune cells, and discovered that Bifidobacterium appeared to be associated with optimized anticancer responses. Bifidobacterium treatment in less sensitive mice enhances tumor suppression and IFNɤ production [53]. Another study then showed that anti-CTLA-4 therapy inhibited tumor development in pathogen-free mice, but not germ-free or antibiotic-treated mice. These findings demonstrated the importance of the microbiota in modifying therapy success [39]. These very first studies found that commensal microbiome species have a role in modifying the therapeutic response of checkpoint inhibitors. Several studies on solid cancer patients have been carried out to assess the microbiome’s effect on ICI responsiveness.

On the other hand, even some classic chemotherapy drugs have effects on the microbiome like cyclophosphamide, which was shown to change the gut microbiota in the small intestine. Cyclophosphamide promotes the enrichment of selected gram-positive bacteria (including Lactobacillus johnsonii, Lactobacillus murinus and Enterococcus hirae) into secondary lymphoid structures, which in return stimulate the growth of unique subsets of “pathogenic” T helper 17 cells and memory Th1 immune responses, eventually contributing to antitumor immune response restoration [40]. Multiple studies on cancer patients have been performed to evaluate the effects of microbiota on ICI. Herein, we highlight clinical studies on various cancer types that evaluated the connection between the microbiome and immune response.

## 5. The Microbiome and ICI Efficacy in Various Solid Malignancies

### 5.1. Melanoma

A phase 1 study on 10 patients with metastatic melanoma who were unresponsive to anti-PD-1 therapy was recently published [54]. Patients were given FMT from two donors who had undergone anti-PD-1 monotherapy and had achieved a complete response. The reported clinical responses included two partial responses and one complete response. The combination of FMT from the complete response donors and anti-PD-1 re-induction in patients with refractory advanced melanoma was safe and efficacious. This approach increased intra-tumoral immune activation in certain patients, translating into objective therapeutic responses. These data support modifying the gut microbiota to overcome immunotherapy resistance [54]. This study demonstrates that microbial intervention, specifically microbial transplantation, can increase the sensitivity of immunotherapy or mitigate the side effects to some degree. However, lacking a control arm is one of the things that makes this study less precise about the context. Further studies are needed to further understand the potential of FMT. Gopalakrishnan et al. performed a study that focused on the differences in gut microbiota diversity and composition between ICI responders (R) and non-responders (NR) in melanoma patients [36]. Researchers used metagenomics analysis to evaluate 43 patients’ fecal samples, 30 R and 13 NR, and found a strong microbiome clustering effect in each group, and α-diversity was much more significant in R than NR [36]. Operational taxonomic unit analysis indicated that patients enriched in Clostridiales/Ruminococcaceae were more likely to respond successfully to PD-1 blocking than those enriched in Bacteroidales. The Faecalibacterium genus (one of the Ruminococcaceae family, Clostridiales order) attracted the researchers’ interest based on the findings of metagenomics studies at all levels. Those with a high Faecalibacterium abundance had a more prolonged, progression-free survival (PFS) (*p* = 0.03) and a lower hazard ratio (HR = 2.92, 95% CI = 1.08–7.89) than patients with a low Faecalibacterium abundance [36].

Furthermore, contrary to the Bacteroidales order, the amount of tumor-infiltrating CD8+ T lymphocytes was favorably associated with the abundance of the Faecalibacterium genus. Patients with Faecalibacterium, Clostridiales, and Ruminococcaceae overrepresentation had more effector T cells in their peripheral blood, whereas patients with Bacteroidales overrepresentation had more Tregs and myeloid-derived suppressor cells. Numerous immunohistochemical studies revealed that patients enriched in Faecalibacterium had higher levels of immune markers, and those findings were supported by fecal microbiota transplantation in mice [36]. These results were further confirmed in a larger cohort (*n* = 132), as both taxa were enriched in responders as well [55]. However, alpha and beta diversity did not show any significant difference between responders and non-responders. The authors suggest the relatively small sample size in the original study as a cause for this discrepancy. In the follow-up study, dietary habits, and probiotics intake, which are both known to affect the microbiome component, have been assessed in relation to the ICI response. Notably, higher dietary fiber was associated with a significant improvement in PFS with melanoma patients on anti-PD-1, with the most benefit reported in patients with an adequate dietary fiber intake and no probiotic use [55].

### 5.2. Lung Cancer

In a study that included 70 Japanese non-small cell lung cancer (NSCLC) patients who were administered anti-PD-1 or anti-PD-L1 therapy, pre-ICI baseline fecal samples showed that in patients who were antibiotics-free, Ruminococcaceae UCG 13 and Agathobacter were enriched in patients with favorable objective response rates (ORR) (achieved a complete response (CR), partial response (PR) or continuous stable disease (SD) for more than 6 months) and a PFS longer than 6 months [56]. In addition, Ruminococcaceae UCG 13 was highly enriched in patients with an overall survival (OS) longer than 12 months. On the other hand, patients who received an antibiotic course (*n* = 16) prior to ICIs had lower alpha diversity (the number of distinguishable taxa) and underrepresentation of Ruminococcaceae UCG 13. Regarding the safety profile, Akkermansia, Lactobacillaceae, and Raoultella were associated with less severe immune-related adverse events in the total sample [56]. In another report, Grenda et al. analyzed 47 stool samples from NSCLC patients who went on to receive anti-PD-1 or anti-PD-L1 in the first or second line. They found that the percentage of Akkermansia was higher in patients achieving SD and PR in comparison to those progressing [57]. Notably, Akkermansia was more enriched in squamous cell carcinoma in comparison to adenocarcinoma [57]. A recent study attempted to assess the microbiome profile using Bronchoalveolar lavage fluid samples in relation to PD-L1 expression in NSCLC patients (*n* = 84) [58]. While alpha and beta diversities did not differ significantly between high and low PD-L1 expression patients, the population of Neissseria was significantly higher in low PD-L1 expression patients [58]. Chau et al. reported on NSCLC patients responding to chemoimmunotherapy, in which Finegoldia was enriched in the nasal microbiome, yet buccal samples showed increased Megasphaera but reduced Actinobacillus in responders [59].

### 5.3. Colorectal Cancer

Forty-two patients with metastatic colorectal cancer who participated in a study had received ≥2 cycles of chemotherapy and were resistant or intolerant to fluorouracil, oxaliplatin, and irinotecan. They were treated with Regorafenib plus Toripalimab (an anti-PD-1). According to the modified toxicity probability interval (mTPI) design, the dose of regorafenib was increased from 80 mg to 120 mg and subsequently reduced to 80 mg. To investigate the relationship between the gut microbiota and treatment effectiveness, conduction of 16S ribosomal RNA (rRNA) sequencing was performed on baseline fecal samples from 32 patients with the greatest clinical response of partial response (PR), stable disease (SD), or progressive disease (PD). Responders (PR or SD; *n* = 11) and non-responders (NRs) (PD; *n* = 21). A comparative investigational analysis revealed that NRs had a significantly higher abundance of Fusobacteriota and a lower abundance of the Proteobacteria phylum. According to the gut microbiome analysis of baseline fecal samples, Fusobacterium had a much higher relative abundance and positive detection rate in NRs than responders. Individuals with a high Fusobacterium abundance had a shorter PFS than those with lower amounts (median PFS = 2.0 vs. 5.2 months; *p* = 0.002) [60]. Lacking a control group for a more accurate comparison is one of the limitations of this study. In another study on CRC patients, Fusobacteria, which is known to be one of the main bacteria associated with poor prognosis, was found to be correlated with lower levels of T cell infiltration and microsatellite instability (MSI), as well as BRAF mutations [61,62,63]. Specifically, F. nucleatum seems to impose its immune suppressive effects in MSI-high tumors, as it was found to correlate inversely with tumor-infiltrating lymphocytes (TIL) in MSI- high tumors, but positively correlated with TILs in MSI-low tumors [64].

### 5.4. Renal Cell Carcinoma (RCC)

A study included 30 patients with RCC who were treatment-naive. Patients were enrolled into two cohorts; one group received nivolumab and ipilimumab, and the other group received the same drug combination plus daily oral CBM588 (a bifidogenic live bacterial product). The results showed a superior median progression-free survival with the nivolumab–ipilimumab plus CBM588 arm compared to the nivolumab–ipilimumab arm. (12.7 versus 2.5 months, hazard ratio (HR) 0.15, 95% CI 0.05–0.47, *p* < 0.001). Even though not statistically significant, patients receiving CBM588 had a greater response rate (58% versus 20%, *p* = 0.06). There was no significant difference in toxicity across the two arms. The results of this study should support the development of future larger studies on this population to validate the findings [65].

### 5.5. Oral Cancer

A phase 1 clinical trial enrolled 25 patients, with 12 in the placebo group (6 healthy people and 6 cancer patients) and 13 in the study group, who were given APG-157 (a botanical medicine containing several polyphenols, including curcumin, produced under the US Food and Drug Administration’s Botanical Drug Development program) [66]. This was given every hour for 3 h (also the same for placebos). Prior to each dose (the 1 h, 2 h, 3 h, and 24 h after treatment doses), blood and saliva samples were taken. Salivary microbial flora analysis in cancer patients revealed a decrease in Bacteroidetes species. RNA and immunofluorescence investigations of a subject’s tumor tissue revealed elevated expression of genes involved in differentiation and T-cell recruitment to the tumor microenvironment. These results indicate that APG-157 treatment may significantly modify the oral microbiota by reducing Bacteroides. Bacteroides were previously linked to colon cancer responses in human studies and mice models in which colonization with Bacteroides increased colon cancer susceptibility. This is thought to be due to its activation of pro-inflammatory cytokines and the upregulation of the WNT signaling pathway. For instance, enterotoxigenic strains of Bacteroides produce the virulence factor B. fragilis toxin which upon expression induces colitis, disrupts E-Cadherin junctions, and induces IL-8 secretion from epithelial colon cells [67]. These alterations might be linked to reduced inflammatory cytokine levels in the saliva, which may help cancer patients to be treated more extensively. The APG-157 study findings provide a statistically significant reduction in the concentrations of inflammatory cytokines and Bacteroides species in salivary cells. T-cell recruitment to the tumor microenvironment was detected in pretreatment and posttreatment tumor samples from cancer patients, suggesting that APG-157 could be used in combination with a checkpoint blockade inhibitor as an adjuvant and support further research development in this space [66].

### 5.6. Hepatobiliary Cancer

A study included 65 patients with advanced hepatobiliary carcinoma who received anti-PD-1 therapy. Among microbiota derived from baseline fecal samples which were followed by continuous sampling the day before each anti-PD-1 infusion, there was a higher abundance of Lachnospiraceae bacterium-GAM79 and Alistipes sp. Marseille-P5997, Ruminococcus calidus, and Erysipelotichaceae bacterium-GAM147 were enriched in the clinical benefit response (CBR) group, and were associated with higher PFS and overall survival (OS) than patients with lower abundance. In contrast, patients with a greater abundance of Veillonellaceae, which was considerably enriched in the non-clinical benefit (NCB) group, had lower PFS and OS [68]. In another study, Shen et al. reported on 36 hepatocellular carcinoma (HCC) patients who were administered anti-PD-1/PD-L1 as monotherapy or in combination with an antiangiogenic agent (bevacizumab or sorafenib). Fecal samples were taken 7 days before ICI initiation and after 8 weeks [69]. There was no difference between responders and non-responders in alpha diversity, richness, or composition of baseline gut microbiome. Only three taxa—Bifidobacterium, Coprococcus, and Acidaminococcus—were higher in patients with disease control, however, their baseline abundance did not correlate with OS [69].

### 5.7. Gastric Cancer

In gastric cancer, H. Pylori is one of the most important microbiota components. It has been classified as a class 1 carcinogen by the World Health Organization [70]. H. Pylori imposes its effects on the composition of gastric microbiota by increasing gastric pH and inducing unique environments for bacterial colonization [70]. In regard to ICIs, Das et al. reported increased gastric epithelial PD-L1 expression with H. Pylori infection as well as decreased CD4+ T cells proliferation, thus identifying those patients as potential responders to anti-PD-L1 therapy [71]. In addition, previous studies have implicated an increase in PD-L1 expression on gastric biopsies of patients with H. Pylori infection. Interestingly, increased PD-L1 expression in gastric cells significantly induced T cell apoptosis [72].

## 6. Role of Antibiotics

Antibiotics influence the gut microbiome by reducing bacterial diversity. Depending on the antibiotic duration, use, and type, restoring the microbiota might take longer than 6 weeks [35,73,74]. Antibiotics, as microbiome-modifying medications, are likely to impact the overall efficacy of immunotherapy [75]. Numerous observational studies suggest that the use of antibiotics may reduce ICI effectiveness. A study investigated the effects of antibiotics administered 2 months before or 1 month after using anti-PD-1/anti-PD-L1. It included 140 non-small-cell lung cancer (NSCLC) patients, 67 renal cancer patients, and 42 urothelial carcinoma patients. The study results revealed that PFS (4.1 vs. 3.5 months; *p* = 0.017) and OS (11.5 vs. 20.8 months; *p* = 0.001) were considerably worse in antibiotic-treated individuals [19]. Additionally, a recent meta-analysis found that using antibiotics before or during ICI administration reduces the OS results, particularly when used just before or after initiating ICIs [76]. A retrospective study revealed that patients with advanced melanoma, non-small-cell lung cancer, and renal cell carcinoma who received an ICI agent with antibiotic usage 2 weeks before and 6 weeks following ICI therapy had lower PFS and OS. This impact increased significantly with cumulative antibiotic use, and may be a result of an antibiotic-induced microbiota imbalance [73]. In a different study, antibiotic treatment at 30 days prior to initiating ICIs negatively impacted the response rate and survival outcome, while the concurrent use of both antibiotics and ICI was not associated with an inferior response rate or decreased OS [77]. A trial by Derosa et al. revealed that immune-related colitis was not seen when fecal samples were enriched in Bacteroidetes and low in Firmicutes. This study also demonstrated a rise in progressive illness and lower PFS and OS among ICI-treated patients on antibiotics [77]. On the other hand, a recent international cohort study, contrary to most previous studies, showed that in 30 days prior to or after the initiation of ICI, antibiotic therapy is associated with improved immunotherapy effectiveness, regardless of disease- and treatment-related variables [78]. Given the conflicting results of various studies, deeper testing and data from larger perspective studies are needed to better evaluate the impact of antibiotics exposure on ICI efficacy.

## 7. Conclusions

**Figure 1:**
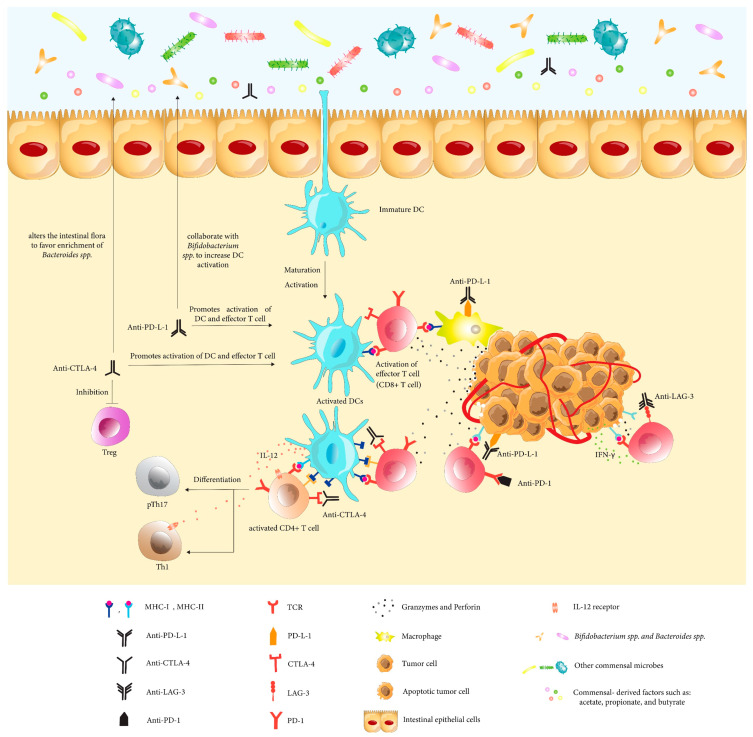
Association between Immunotherapy and Microbiome. Microbiome components (specific Bacteroides spp and Burkholderiales) such as short-chain fatty acids (SCFAs), mainly acetate propionate and butyrate, improve the efficiency of CTLA-4 blockade by promoting tumor control through stimulating Th1 immune responses during anti-CTLA-4 therapies. Anti-CTLA-4 indirectly modifies the gut flora to promote Bacteroides species enrichment, possibly by enhancing epithelial barrier breakdown. Consequently, these enriched species boost the activation and maturation of dendritic cells (DCs), which provide tumor antigens to enhance the recruitment and activity of T lymphocytes, inducing pTh17 and Th1 differentiation. In addition, anti-CTLA-4 blocks the immunosuppressive function of regulatory T cells (Tregs). Anti-PD-L1 treatment depends on the presence of important genera in the host, specifically Bifidobacterium, which promotes DCs activation and antitumor T cell responses. These processes spread systemically and suppress cancer cells through augmenting Th1 and CD8+ T cells and upregulating IFN-γ and Granzyme B while on anti-CTLA-4 and anti-PD-1/PD-L1 therapy.

The studies discussed in this review highlight the potential relationship between gut microbiota and the immune system (Figure 1). Cancer immunotherapy is currently one of the bases of cancer treatment. The impact of gut microbiota on tumor growth and its modulatory effect on cancer therapeutics, especially immunotherapy, has increasingly been recognized as genomics and metabolomics technologies have matured. The specific role the microbiome plays on immunotherapy response and toxicity factors is still not very well established and/or understood. Certain variables, like the balance of specific intestinal microbiota species, have defined poor or better survival outcomes in ICIs studies. Those should be further tested as potential predictive/prognostic biomarkers for ICIs. Several prospective clinical trials are currently ongoing (Table 1) and further understanding of microbiome ICIs crosstalk is eagerly awaited.

Microbial intervention, such as microbial transplantation through FMT, may boost the effectiveness of immunotherapy and potentially minimize immunotherapy adverse reactions. Notably, gut microbiome bacteria have four significant phyla: Firmicutes, Bacteroidetes, Proteobacteria, and Fusobacter. Numerous issues must be addressed, including whether species, signature, or metabolites are the most critical immunomodulatory components to enable FMT fine-tuning to address those factors, and whether FMT or probiotic therapy is more effective to modulate cancer immunotherapeutic responses. More research on bacterial usage in such settings is needed to increase the granularity in this research space and further assess its effectiveness and safety in cancer patients.

## Figures and Tables

**Table 1:** Ongoing Clinical Trials * Testing Microbiome Impact on the Efficacy of Immunotherapy.

	Study Title	Condition	Intervention	Outcome	Participant (*n*)	Estimated Study Completion Date	Immunotherapy
1	Role of Microbiome in the Realm of Immune-Checkpoint Inhibitor Induced GI Complications in Cancer Population	Melanoma Lung Cancer	Fecal Microbiota Transplantation (FMT)	The difference in stool microbiome pattern, incidence of adverse events (AE) of fecal microbiota transplantation	800	30 January 2023	Infliximab
2	Intestinal Microbiome Modification with Resistant Starch in Patients Treated with Dual Immune Checkpoint Inhibitors	Solid Tumors	Potato Starch (Bob’s Red Mill^®^, Milwaukie, OR, USA)	Number of patients able to adhere to resistant starch (RS) supplement schedule, adverse events (SAEs) attributable to ICI therapy, Occurrence of unanticipated serious adverse events (SAEs)	12	September 2022	Dual ICI regimens
3	CBM588 in Combination with Nivolumab and Cabozantinib for the Treatment of Advanced or Metastatic Kidney Cancer	RCC	Clostridium butyricum CBM 588 Probiotic Strain	The effects Of CBM588 On Gut Microbiome in Patients With RCC	30	30 November 2023	Nivolumab
4	A Phase II Clinical Trial of Anti-PD-1 mAb Therapy Alone or With Metabolic Modulators to Reverse Tumor Hypoxia and Immune Dysfunction in Solid Tumor Malignancies	Melanoma, NSCLC Hepatocellular Carcinoma Urothelial Cancer Gastric Adenocarcinoma HNSCC Esophageal Adenocarcinoma Microsatellite instability-high Solid Malignant Tumor	Metformin Rosiglitazone	PFS, OS, Best overall response, Oral and Stool Microbiome, Adverse Events,	108	December 2027	Nivolumab or Pembrolizumab

* This information is available on clinicaltrial.gov (accessed on 1 March 2022).

## Data Availability

Not applicable.
